# Editorial: The association between the nervous system and the stomatognathic system: from etiology to diagnosis and treatment of orofacial pain

**DOI:** 10.3389/fneur.2026.1824740

**Published:** 2026-04-10

**Authors:** Liliana Szyszka-Sommerfeld, Magdalena Sycińska-Dziarnowska, Gianrico Spagnuolo, Krzysztof Woźniak

**Affiliations:** 1 Laboratory for Propaedeutics of Orthodontics and Facial Congenital Defects, Chair of Maxillofacial Orthopaedics and Orthodontics, Pomeranian Medical University in Szczecin, Szczecin, Poland; 2 Department of Maxillofacial Orthopaedics and Orthodontics, Pomeranian Medical University in Szczecin, Szczecin, Poland; 3 Department of Neurosciences, Reproductive and Odontostomatological Sciences, University of Naples “Federico II”, Naples, Italy

**Keywords:** craniofacial cavities, etiology, nervous system, orofacial pain, pain, stomatognathic system

Pain is a complex biopsychosocial phenomenon that results from the interaction of multiple neuroanatomic and neurochemical systems with a range of cognitive and affective processes (1). According to the International Association for the Study of Pain, the definition of pain is as follows: “*Pain is an unpleasant sensory and emotional experience associated with actual or potential tissue damage, or described in terms of such damage*” (2). Pain, whether related to injured tissue, inflammation or functional impairment, is mediated by processing in the nervous system (3).

Orofacial pain (OFP) is the most common reason for patients to visit the dentist, but this area is complex, and pain can be related to hard and soft tissues of the head, face, oral cavity, or nervous system dysfunction. Disorders associated with orofacial pain include but are not limited to temporomandibular muscle and joint disorders, jaw movement disorders, neuropathic and neurovascular pain disorders, headache, and sleep disorders (4). In general, dental pain is the most common type of orofacial pain. On the other hand, temporomandibular disorder (TMD) is a set of painful conditions affecting the orofacial region that is prevalent and constitute the most frequent type of non-dental pain complaint in the maxillofacial area (5). The origins of TMD pain are complex, with various factors contributing to its development or persistence. These factors can include biological aspects like hormones, genetic predispositions, physical trauma, systemic disorders, occlusal interferences, parafunctional habits, and psychological influences like stress levels and emotional responses, social, and environmental variables (6, 7). Due to the multiple factors that contribute to the development of this condition, its accurate diagnosis can be difficult (5). Another reason of OFP is pain associated with orthodontic tooth movement which is accompanied by inflammatory responses in the periodontal ligaments and modulation of neural activity in the central nervous system (8).

This Research Topic aimed to address various aspects of the association between the nervous system and the stomatognathic system, considering the etiology, diagnosis, and treatment of orofacial pain conditions. Eleven articles were included in this Research Topic.

Six articles dealt with pain related to temporomandibular disorder (TMD). One of these studies analyzed the current status of TMD imaging education based on a synthesis of literature and educational practices (Zhao et al.). Two articles concerned the association between pain-related TMD and malocclusion (Gałczyńska-Rusin, Maciejewska-Szaniec et al.; Szyszka-Sommerfeld et al.). One study assessed the occurrence of oral parafunctions, TMD pain, and headaches in Polish patients undergoing orthodontic treatment (Gałczyńska-Rusin, Szyszka-Sommerfeld et al.), and another study concerned oral behaviors in Chinese temporomandibular disorder patients (Liu et al.). One study determined the relationship between salivary/blood concentrations of oxidative/nitrosative stress biomarkers and biopsychosocial findings in patients with temporomandibular disorder-myofascial pain with referral (Kuć et al.).

Three articles concern pain associated with orthodontic tooth movement. Two of them covered the topic of the association between patients' personality traits and pain during orthodontic treatment with fixed appliances (Lorek, Jarzabek, Sycińska-Dziarnowska, Gołab, Cichocka et al.; Lorek, Jarzabek, Sycińska-Dziarnowska, Gołab, Krawczyk et al.). One study examined the impact of low-level laser therapy on orthodontic pain (Jagła et al.).

A single article concerned trigeminal somatosensory evoked potentials for intraoperative monitoring and prognostic prediction in microvascular decompression for trigeminal neuralgia (Zhang et al.). Another study aimed to assess the impact of photobiomodulation on pain control in maxillary permanent molars affected by MIH (Olszewska et al.).

Published articles confirm the multifactorial and complex etiology of orofacial pain conditions. Given the numerous and diverse potential origins of orofacial pain, a comprehensive assessment of the situation is essential to establish the most appropriate diagnostic pathway and to ensure optimal clinical and therapeutic management.
